# Hemodynamics combined with inflammatory indicators exploring relationships between ischemic stroke and symptomatic middle cerebral artery atherosclerotic stenosis

**DOI:** 10.1186/s40001-023-01344-8

**Published:** 2023-09-26

**Authors:** Xiao-Bing Wu, Yi-Ao Liu, Li-Xin Huang, Xin Guo, Wang-Qing Cai, Bin Luo, Sheng-Wen Wang

**Affiliations:** 1 Department of Neurosurgery, Sun Yat-Sen Memorial Hospital, Sun Yat-Sen University, 107 Yanjiang West Road, Guangzhou, 510120 Guangdong China; 2 Department of Neurosurgery, The Eighth Affiliated Hospital, Sun Yat-Sen University, 3025 Shennan Middle Road, Shenzhen, 518033 China; 3 Department of Neurosurgery, The Third Affiliated Hospital, Sun Yat-Sen University, Guangzhou, China

**Keywords:** Intracranial atherosclerotic stenosis, Plaque enhancement, Plaque burden, Hemodynamics, Wall shear stress, Lymphocyte–monocyte ratio

## Abstract

**Background and purpose:**

Intracranial atherosclerotic stenosis (ICAS) is a major cause of ischemic stroke, and high-resolution vessel wall imaging (HR-VWI) can be used to assess the plaque characteristics of ICAS. This study combined HR-VWI, hemodynamics, and peripheral blood inflammatory indicators to investigate the role of these factors in symptomatic intracranial atherosclerotic stenosis (sICAS) and their inter-relationships.

**Methods:**

Patients diagnosed with atherosclerotic middle cerebral artery stenosis were recruited retrospectively from June 2018 to July 2022. Plaque enhancement was qualitatively and quantitatively analyzed, and the degree of plaque enhancement was graded according to the plaque-to-pituitary stalk contrast ratio (CR). Computational fluid dynamics models were constructed, and then hemodynamic parameters, including wall shear stress (WSS) and pressure ratio (PR), were measured and recorded. Univariate and multivariable analyses were performed to identify factors that can predict sICAS. In addition, the correlation analysis between the plaque characteristics on HR-VWI, hemodynamic parameters, and peripheral blood inflammatory indicators was performed to investigate the interrelationships between these factors.

**Results:**

Thirty-two patients were included. A higher proportion of plaque enhancement, maximum WSS, and WSS ratio (WSSR) were significantly associated with sICAS. The multiple logistic regression analysis showed that only the WSSR was an independent risk factor for sICAS. The correlation analysis revealed that both the CR and plaque burden showed linear positive correlation with the WSSR (*R* = 0.411, *P* = 0.022; *R* = 0.474, *P* = 0.007, respectively), and showed linear negative correlation with the lymphocyte to monocyte ratio (*R* = 0.382, *P* = 0.031; *R* = 0.716, *P* < 0.001, respectively).

**Conclusions:**

The plaque enhancement and WSSR were significantly associated with sICAS, WSSR was an independent risk factor for sICAS. Plaque enhancement and plaque burden showed linear correlation with the WSSR and lymphocyte-to-monocyte ratio (LMR). Hemodynamics and inflammation combined to promote plaque progression.

**Supplementary Information:**

The online version contains supplementary material available at 10.1186/s40001-023-01344-8.

## Introduction

Intracranial atherosclerotic stenosis (ICAS) is one of the most common causes of ischemic stroke and can lead to substantial disability and death [1–4]. ICAS accounts for up to half of all ischemic stroke cases in East Asian populations, particularly among Chinese [5]. The CASSISS study showed that the stroke and mortality rates at 3 years in patients with sICAS remained nearly 18% after standard medical therapy, so there was significant clinical value in searching for the etiology of plaque instability [5]. The current challenge is not only to detect the presence of ICAS plaques but also to identify unstable plaques. The development mechanism of ICAS remains unknow, thus investigating and understanding this mechanism will help develop therapeutic strategies to prevent the formation of ICAS plaques and treat unstable plaques.

Inflammation and hemodynamics are believed to play important roles in the development and progression of atherosclerotic plaques. In fact, pathological studies have revealed that ICAS plaques are characterized by inflammatory infiltration and increased infiltration of a large number of both T lymphocytes and macrophages into plaques is associated with infarction [6, 7]. The development of neuroimaging techniques, such as high-resolution vessel wall magnetic resonance (MR) imaging (HR-VWI), has allowed the detection of instability in ICAS plaques, and gadolinium enhancement on HR-VWI has been used as an imaging marker of ICAS plaque instability [8, 9]. Other pathological studies have also shown that gadolinium enhancement of carotid plaques was associated with vulnerable plaque phenotypes and was related to inflammation [10, 11], which showed that gadolinium enhancement may be an inflammatory manifestation of atherosclerotic plaque. In addition, our previous study found that the lymphocyte-to-monocyte ratio (LMR), a systemic inflammatory biomarker, was independently associated with symptomatic ICAS (sICAS) [12]. Recent studies have shown that wall shear stress (WSS) may be involved in the formation of atherosclerotic lesions and the risk of subsequent ischemic events [13–15]. Overall, hemodynamic assessment plays a significant role in understanding the pathophysiology of ICAS and guiding treatment decisions. Ongoing researches aims to further refine these assessments by combining multiple imaging modalities, developing advanced computational models, and investigating novel biomarkers. The ultimate goal is to improve risk stratification, enhance therapeutic interventions, and prevent stroke recurrence in individuals with ICAS [14, 15]. However, it is unclear whether hemodynamics and inflammation play a role in sICAS. The aim of this study was to investigate the role and relationship of inflammation and hemodynamics in sICAS using a combination of HR-VWI, peripheral blood inflammatory indicators and hemodynamic analysis to provide a theoretical basis for further research into the pathophysiology of ICAS and secondary stroke prevention in patients with ICAS.

## Materials and methods

### Study population and data collection

The data from 72 patients with atherosclerotic middle cerebral artery (MCA) stenosis admitted to Sun Yat-sen Memorial Hospital between June 2018 and July 2022 and who underwent HR-VWI were selected from our institution’s database. The results of laboratory tests and clinical data, including age, sex, current smoking and drinking status, hypertension, and diabetes, were recorded.

The inclusion criteria were as follows: (1) age > 18 years, (2) HR-VWI images had no artifacts, and (3) the patient underwent digital subtraction angiography (DSA), MR angiography (MRA) or computed tomography angiography (CTA) that confirmed MCA stenosis*.* The exclusion criteria were as follows: (1) a more than 50% stenosis of the ipsilateral internal carotid artery, (2) recent cardiocerebrovascular disease, such as cerebral hemorrhage or subarachnoid hemorrhage, coronary heart disease, and atrial fibrillation, (3) history of autoimmune disease, (4) acute or chronic infection, (5) known malignancies, (6) blood disease or serious systemic disease, and (7) surgical history within the last 6 months. The study was approved by the Medical Ethics Committee of Sun Yat-sen Memorial Hospital of Sun Yat-sen University.

### Laboratory data

Blood samples from all patients collected within 24 h after admission were subjected to complete blood cell count analysis and biochemical examinations. The white blood cell count (WBC), neutrophil count (NEUT), lymphocyte count (LYM), monocyte count (MONO), platelet count (PLT), blood glucose (GLU), and blood lipids were recorded. The neutrophil-to-lymphocyte ratio (NLR), lymphocyte-to-monocyte ratio (LMR), platelet-to-lymphocyte ratio (PLR) and systemic immune-inflammation index (SII) were calculated as follows: NLR = NEUT/LYM, LMR = LYM/MONO, PLR = PLT/LYM, and SII = PLT*NEUT/LYM.

### Imaging examinations

#### Computer tomography angiography (CTA)

CTA was performed with a 128-slice Siemens spiral CT scanner (Siemens Healthcare AG, Erlangen, Germany). The parameters are listed in the Additional file 1.

#### High resolution vessel wall magnetic resonance imaging (HR-VWI)

MR imaging (MRI) was collected with an Achieva TX 3.0 T MRI scanner (Philips Healthcare, Best, the Netherlands) equipped with a 32-channel head coil. Conventional cerebral MRI, MRA, diffusion-weighted MRI (DWI), and HR-VWI of each patient were performed. The technical parameters used for the HR-VWI were as previously described [12] and are listed in Additional file 1.

### Image analyses

The degree of stenosis was calculated using the following equation: [1 − (Dstenosis/Dnormal)] × 100% [16].

**Fig. 1:**
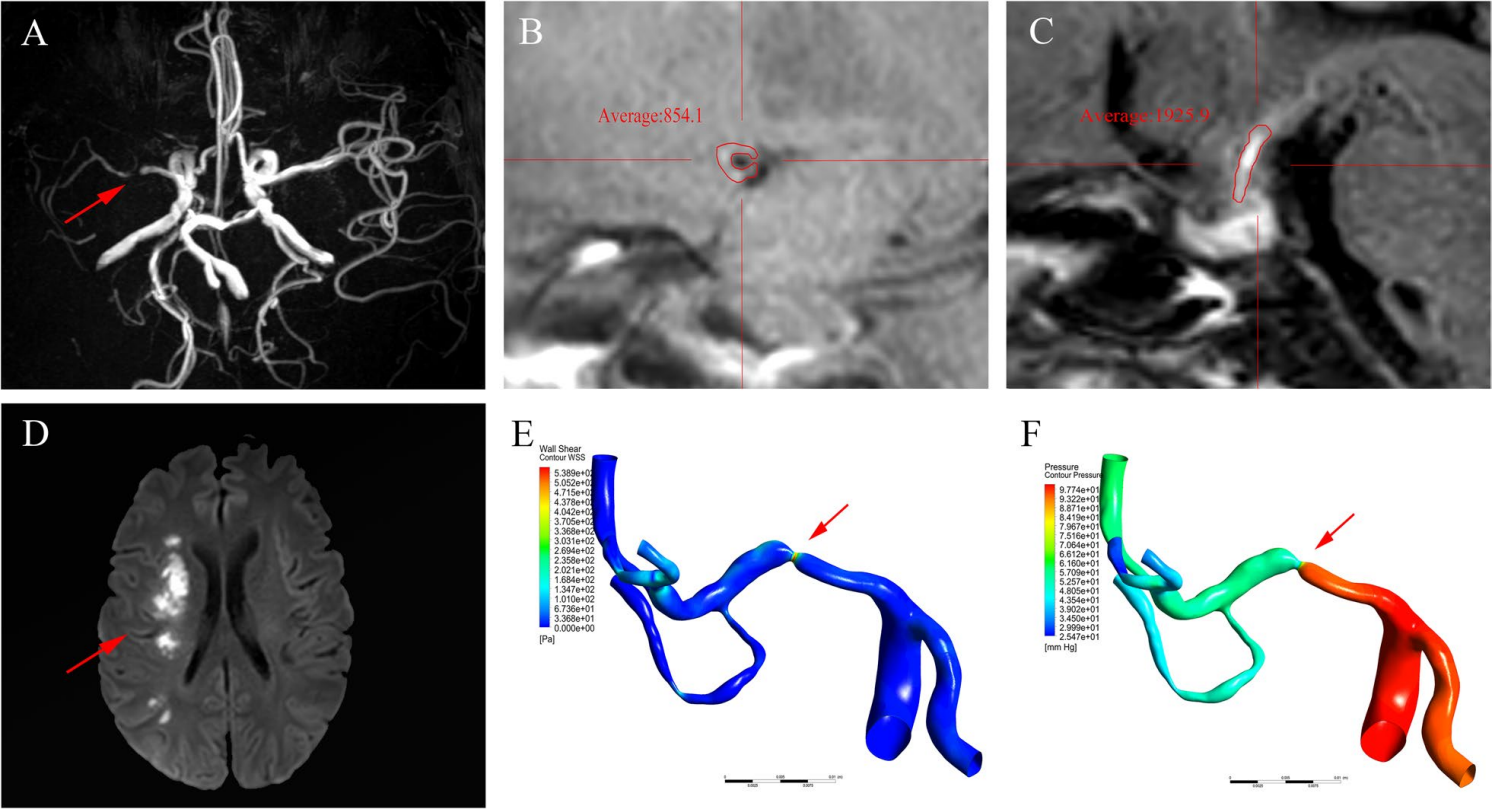
Adult male with severe stenosis of right middle cerebral artery (R-MCA). **A** R-MCA stenosis in MRA; **B**, **C** mean signal value of R-MCA plaque and pituitary stalk on post-contrast HR-VWI; **D** DWI showed acute infarction in the right cerebral hemisphere; **E**, **F** show the results of WSS and pressure contour maps, respectively

Plaque enhancement was qualitatively and quantitatively measured as previously described [12] (Fig. 1). Plaque enhancement was assessed according to the pre- and post-contrast HR-VWI T1-weighted sequences. Images were independently reviewed by two experienced raters who were only informed the stenosis location, but not aware of the clinical data, and determined whether the plaque enhancement had occurred. Disagreements were resolved by consensus. The plaque-to-pituitary stalk contrast ratio (CR) was used as the degree of plaque enhancement and calculated using the following equation: CR = SIplaque/SIstalk, and the mean CR was ultimately calculated by averaging [12].

The conventional MRI and DWI sequences were performed, then the location of an infarction was determined. Lesions were classified as symptomatic or asymptomatic according to the brain infarct on the DWI and/or whether there were signs of neurological deficits during the ischemic events [12].

### Computational fluid dynamics (CFD) procedure

*CFD modeling* Two neurologists, blinded to clinical information, used a personal work station to process the source images of time-of-flight MRA (TOF-MRA) and CTA in the standard Digital Imaging and Communication in Medicine (DICOM) format. These data sets were imported into Materialise's Interactive Medical Image Control System (MIMICS, Version 21.0) software (Materialise NV, Leuven, Belgium) for segmentation and reconstruction of 3D vascular models, and then manually examined and refined by neurologist in Geomagic Studio (Version 14.0) software (Geomagic, Research Triangle Park, NC, USA) to produce a relatively smooth 3D vascular model with ICA, MCA M1–M2 and ACA A1 segments. A mesh was performed using the Ansys Fluent meshing (Version 2020R1) software (Ansys Inc., Canonsburg, PA, USA) to create a polyhedral vessel surface and within the vessel lumen, with the maximal element size of 0.8 and minimal element size of 0.1. In addition, five boundary layers were set, the thickness of the first layer was 0.01 mm, and the layer-by-layer growth rate was 1.2. A better result was obtained for each case by keeping the maximum skewness of the overall mesh quality to less than 0.6 [17, 18].

The CFD simulation was performed using The Ansys Fluent software, treating blood as a viscous and incompressible Newtonian fluid (*ρ* = 1060 kg/m^3^, *μ* = 0.0035 kg m^−1^ s^−1^), flowing in a laminar flow mode with rigid vessel wall and no-slip boundary condition. The operating condition was set to a normal intracranial pressure of 10 mmHg for all the cases. The boundary conditions of the CFD model included the inlet condition at the internal carotid artery, which was applied an average pressure of 110 mmHg, and the outlet condition at the distal cerebral artery with the mass flow outlets based on the mean flow velocities from a population-based study [19–21]. We selected the coupled pressure–velocity coupling method in steady-state blood flow simulation, achieving convergence when the residual of continuity was less than 10^–4^.

*Hemodynamic analysis* Post-processing was performed in the Ansys CFD-post software to extract and measure translesional pressure ratio (PR), WSSmax and WSS ratio (WSSR) (Fig. 1). Translesional PR was defined as the ratio of the mean pressures at the poststenotic and prestenotic arterial segments. The WSSR was defined as the ratio of the mean WSS at the stenotic throat and prestenotic arterial segment [20].

### Statistical analysis

The SPSS 23.0 software (IBM Corporation, Armonk, NY, USA) was used for the statistical analysis of the data. The patients with atherosclerotic MCA stenosis were divided into a plaque enhancement group and a non-enhancement group, and were further divided into a symptomatic group and an asymptomatic group according to whether the plaque was symptomatic. Continuous variable data were subjected to a normal distribution test. Variables with normal distribution are presented as the mean ± standard deviation and were compared using Student’s *t* test for comparison between two groups, while variables with non-normal distribution are presented as medians and were compared using the Mann–Whitney *U* test for comparisons between groups. In addition, categorical variables are expressed as the number of cases and percentages and were compared using Fisher's exact or chi-square test for comparisons between groups.

Multivariate logistic regression analyses with forward condition were performed to determine which factors were independent risk factors for atherosclerotic MCA stenosis with plaque enhancement and symptomatic stenosis after adjusting for variables with *P* < 0.05 in the univariate comparisons. Furthermore, correlation analysis was performed to determine the associations between the plaque characteristics with hemodynamic parameters and inflammatory markers in peripheral blood. A *P* value < 0.05 was considered statistically significant.

## Results

### Patient characteristics

A total of 32 adult patients were included in the present study, their mean age was 57.1 ± 10.0 years, and 17 (53.1%) were male. The clinical characteristics and the percentage of patients with each of the characteristics were as follows: hypertension (62.5%), diabetes (34.4%), smoking (31.3%), and drinking (15.6%) (Table 1). In 17 (53.1%) patients lesions were symptomatic, and in 15 (46.9%) asymptomatic. In 21 (65.6%) patients the MCA stenosis lesions showed plaque enhancement on HR-VWI, while in 11 (34.4%) patients showed no enhancement. The inter-reader agreement for occurrence of plaque enhancement was excellent (κ = 0.862).

**Table 1:** Characteristics of MCA atherosclerotic plaques with and without symptoms

	Total (*n* = 32)	Symptomatic stenosis (*n* = 17)	Asymptomatic stenosis (*n* = 15)	*P* value
Age	57.1 ± 10.0	57.2 ± 9.9	57.0 ± 10.5	0.961
Sex (male)	17 (53.1%)	10 (58.8%)	7 (46.7%)	0.492
BMI	24.5 ± 3.6	23.1 ± 3.0	25.9 ± 3.7	0.026
Hypertension	20 (62.5%)	12 (70.6%)	8 (53.3%)	0.314
Diabetes	11 (34.4%)	7 (41.2%)	4 (26.7%)	0.388
Smoking	10 (31.3%)	4 (23.5%)	6 (40.0%)	0.316
Drinking	5 (15.6%)	3 (17.6%)	2 (13.3%)	0.737
*Degree of stenosis*				0.570
70–99%	20 (62.5%)	12 (70.6%)	8 (53.3%)	
50–69%	9 (28.1%)	4 (23.5%)	5 (33.3%)	
< 50%	3 (9.4%)	1 (5.9%)	2 (13.3%)	
WBC (× 10^9^/L)	7.2 ± 1.6	7.3 ± 1.6	7.2 ± 1.7	0.937
LYM (× 10^9^/L)	2.0 ± 0.6	2.0 ± 0.7	2.1 ± 0.6	0.517
NEUT (× 10^9^/L)	4.4 ± 1.3	4.5 ± 1.3	4.3 ± 1.4	0.749
MONO (× 10^9^/L)	0.5 ± 0.2	0.5 ± 0.2	0.6 ± 0.2	0.842
Platelets (× 10^9^/L)	245.5 ± 46.8	248.2 ± 45.9	242.5 ± 49.1	0.737
GLU(mmol/L)	5.5 (3.9–16.4)	5.6 (3.9–15.6)	5.1 (4.2–16.4)	0.100
Cholesterol (mmol/L)	4.9 ± 1.4	4.8 ± 1.7	5.1 ± 1.1	0.517
Triglyceride (mmol/L)	1.6 (0.6–4.3)	1.6 (0.6–3.9)	1.4 (0.8–4.3)	0.428
HDL (mmol/L)	1.1 ± 0.3	1.0 ± 0.3	1.2 ± 0.3	0.092
LDL (mmol/L)	3.2 ± 1.1	3.1 ± 1.4	3.3 ± 0.8	0.736
Apolipoprotein E (mg/L)	36.6 ± 11.0	35.6 ± 11.9	37.5 ± 10.2	0.662
hsCRP (mmol/L)	1.7 (0.2–26.9)	1.4 (0.2–18.3)	2.0 (0.3–26.9)	0.571
NLR	2.4 ± 1.1	2.6 ± 1.1	2.2 ± 1.0	0.418
LMR	4.1 ± 1.5	3.9 ± 1.6	4.2 ± 1.5	0.680
LMR ≤ 4.0 (%)	17 (53.1%)	11 (64.7%)	6 (40.0%)	0.162
PLR	133.4 ± 49.6	140.2 ± 49.9	125.8 ± 49.7	0.420
SII	603.1 ± 319.8	649.9 ± 315.5	550.1 ± 282.1	0.387
Plaque burden	0.8 ± 0.1	0.8 ± 0.1	0.8 ± 0.1	0.186
Plaque area (mm^2^)	13.2 ± 6.9	14.3 ± 7.3	12.0 ± 6.5	0.360
Plaque enhancement	21 (65.6%)	14 (82.4%)	7 (46.7%)	0.034
CR	0.54 ± 0.14	0.56 ± 0.12	0.52 ± 0.13	0.486
WSSmax (Pa)	258.1 ± 142.0	324.0 ± 146.8	184.5 ± 93.6	0.003
WSSR	85.0 ± 54.2	112.8 ± 51.3	53.5 ± 38.8	0.001
PR	0.7 ± 0.2	0.6 ± 0.2	0.7 ± 0.2	0.004
Hospitalization days	10.4 ± 5.4	12.7 ± 6.0	7.7 ± 3.2	0.008

*MCA* middle cerebral arterial, *BMI* body mass index, *WBC* white blood cells, *LYM* lymphocyte counts, *NEUT* neutral counts, *MONO* monocyte counts, *CRP* C-reactive protein, *NLR* neutrophil-to-lymphocyte ratio, *LMR* lymphocyte-to-monocyte ratio, *PLR* platelet-to-lymphocyte ratio, *SII* systemic immune-inflammation index, *CR* the plaque-to-pituitary stalk contrast ratio, *WSSmax* the maximum wall shear stress, *WSSR* WSS ratio, *PR* pressure ratio

### The WSSR as an independent predictor for symptomatic stenosis

Univariate comparisons of the clinical, radiologic and laboratory variables showed that the rate of MCA plaque enhancement (82.4% vs. 46.7%, *P* = 0.034) and the WSSmax, WSSR, and hospitalization days of the sICAS group were significantly larger than those of the asymptomatic ICAS group (*P* = 0.003, *P* = 0.001, *P* = 0.008, respectively). The level of the PR of the symptomatic ICAS patients was significantly lower than those of asymptomatic ICAS patients (*P* = 0.004) (Table 1). The multivariate logistic regression analysis revealed that the WSSR was the only statistically significant independent predictor of ischemic stroke symptoms (OR: 1.026, 95% CI 1.007–1.046, *P* = 0.007) (Table 2).

**Table 2:** Multiple logistic regression analysis for symptomatic stenosis using forward method

Variable	Odds ratio	95% Confidence interval	*P* value
WSSR	1.026	1.007–1.046	0.007

*WSSR* WSS ratio

### The WSSR as an independent predictor of plaque enhancement

Patients with plaque enhancement were more likely to have a larger WSSR, more hospitalization days (*P* = 0.001,* P* = 0.031, respectively) and lower levels of LMR (*P* = 0.006) (Table 3). These variables (*P* ≤ 0.05) were subsequently entered into the forward conditional multiple logistic regression model to determine the risk factors for plaque enhancement. The results showed that the WSSR (OR 1.034, 95% CI 1.009–1.060, *P* = 0.007) was independently associated with plaque enhancement (Table 4).

**Table 3:** Characteristics of MCA atherosclerotic plaques with and without enhancement

	Total (*n* = 32)	Enhancement (*n* = 21)	Nonenhancement (*n* = 11)	*P* value
Age	57.1 ± 10.0	59.4 ± 8.5	52.6 ± 11.4	0.066
Sex (male)	17 (53.1%)	11 (52.4%)	6 (54.5%)	0.907
BMI	24.5 ± 3.5	24.6 ± 3.5	24.2 ± 4.0	0.789
Hypertension	20 (62.5%)	14 (66.7%)	6 (54.5%)	0.501
Diabetes	11 (34.4%)	7 (33.3%)	4 (36.4%)	0.864
Smoking	10 (31.3%)	7 (33.3%)	3 (27.3%)	0.725
Drinking	5 (15.6%)	4 (19.0%)	1 (9.1%)	0.461
*Degree of stenosis*				0.053
70–99%	20 (62.5%)	16 (76.2%)	4 (36.4%)	
50–69%	9 (28.1%)	3 (14.3%)	6 (54.5%)	
< 50%	3 (9.4%)	2 (9.5%)	1 (9.1%)	
WBC (× 10^9^/L)	7.2 ± 1.6	7.4 ± 1.6	7.0 ± 1.8	0.518
LYM (× 10^9^/L)	2.0 ± 0.6	1.9 ± 0.6	2.2 ± 0.7	0.182
NEUT (× 10^9^/L)	4.4 ± 1.3	4.6 ± 1.3	4.1 ± 1.4	0.299
MONO (× 10^9^/L)	0.5 ± 0.2	0.6 ± 0.2	0.5 ± 0.3	0.550
Platelets (× 10^9^/L)	245.5 ± 46.8	248.5 ± 46.5	239.8 ± 50.8	0.627
GLU(mmol/L)	6.4 ± 2.9	6.4 ± 2.7	6.3 ± 3.5	0.901
Cholesterol (mmol/L)	4.9 ± 1.4	4.8 ± 1.7	5.2 ± 0.9	0.536
Triglyceride (mmol/L)	1.9 ± 0.9	1.8 ± 0.8	2.0 ± 1.1	0.583
HDL (mmol/L)	1.1 ± 0.3	1.0 ± 0.3	1.1 ± 0.3	0.229
LDL (mmol/L)	3.2 ± 1.1	3.1 ± 1.3	3.3 ± 0.7	0.697
Apolipoprotein E (mg/L)	36.6 ± 11.0	34.5 ± 10.7	40.6 ± 10.7	0.140
hsCRP (mmol/L)	1.7 (0.2–26.9),	1.4 (0.2–18.3),	2.1 (0.6–26.9),	0.331
NLR	2.4 ± 1.1	2.6 ± 1.1	2.0 ± 1.0	0.118
LMR	4.1 ± 1.5	3.5 ± 1.0	5.0 ± 1.9	0.006
LMR ≤ 4.0 (%)	17 (53.1%)	14 (66.7%)	3(27.3%)	0.034
PLR	133.4 ± 49.6	143.0 ± 52.2	115.1 ± 40.0	0.133
SII	603.1 ± 319.8	668.6 ± 335.1	478.2 ± 257.4	0.111
Plaque burden	0.8 ± 0.1	0.8 ± 0.1	0.7 ± 0.1	0.056
Plaque area (mm^2^)	13.2 ± 6.9	14.2 ± 6.8	11.3 ± 7.0	0.257
WSSmax (Pa)	258.1 ± 142.0	276.8 ± 141.7	222.5 ± 142.1	0.312
WSSR	85.0 ± 54.2	107.0 ± 49.3	43.1 ± 36.3	0.001
PR	0.7 ± 0.2	0.7 ± 0.2	0.7 ± 0.3	0.820
Hospitalization days	10.4 ± 5.4	11.9 ± 5.6	7.6 ± 3.9	0.031

*MCA* middle cerebral arterial, *BMI* body mass index, *WBC* white blood cells, *LYM* lymphocyte counts, *NEUT* neutral counts, *MONO* monocyte counts, *CRP* C-reactive protein, *NLR* neutrophil-to-lymphocyte ratio, *LMR* lymphocyte-to-monocyte ratio, *PLR* platelet-to-lymphocyte ratio, *SII* systemic immune-inflammation index, *CR* the plaque-to-pituitary stalk contrast ratio, *WSSmax* the maximum wall shear stress, *WSSR* WSS ratio, *PR* pressure ratio

**Table 4:** Multiple logistic regression analysis for MCA atherosclerotic stenosis with plaque enhancement using forward method

Variable	Odds ratio	95% Confidence interval	*P* value
WSSR	1.034	1.009–1.060	0.007

*WSSR* WSS ratio

### *The WSSR and the CR were found to be significantly associated with LMR* ≤ *4.0*

**Fig. 2:**
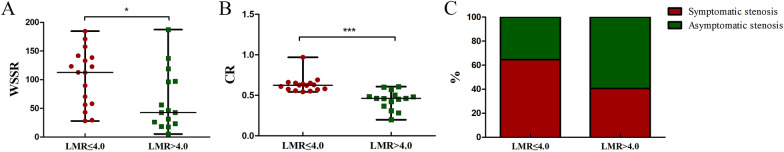
**A** WSSR was compared between the group of LMR ≤ 4.0 and LMR > 4.0 (104.3 ± 49.3 vs. 63.1 ± 52.6, *P < 0.05). **B** CR was compared between the group of LMR ≤ 4.0 and LMR > 4.0 (0.63 ± 0.10 vs. 0.44 ± 0.12, ***P < 0.001). **C** Proportion of symptomatic stenosis was compared between the group of LMR ≤ 4.0 and LMR > 4.0 (64.7% vs*.* 40%, *P* = 0.162)

We compared the WSSR and CR between the LMR ≤ 4.0 group and the LMR > 4.0 group, the results revealed that the WSSR (104.28 ± 49.32 vs. 63.16 ± 52.65, *P* = 0.030; Fig. 2A) and CR (0.63 ± 0.10 vs. 0.44 ± 0.12, *P* < 0.001; Fig. 2B) of the LMR ≤ 4.0 group were significantly larger than that of the LMR > 4.0 group. In addition, we found that the proportion of sICAS in the LMR ≤ 4.0 group was higher than that in the LMR > 4.0 group (64.7% vs. 40%), but the difference was not statistically significant (*P* = 0.126) (Fig. 2C).

### Linear correlation between the plaque characteristics with both the WSS and LMR

**Fig. 3:**
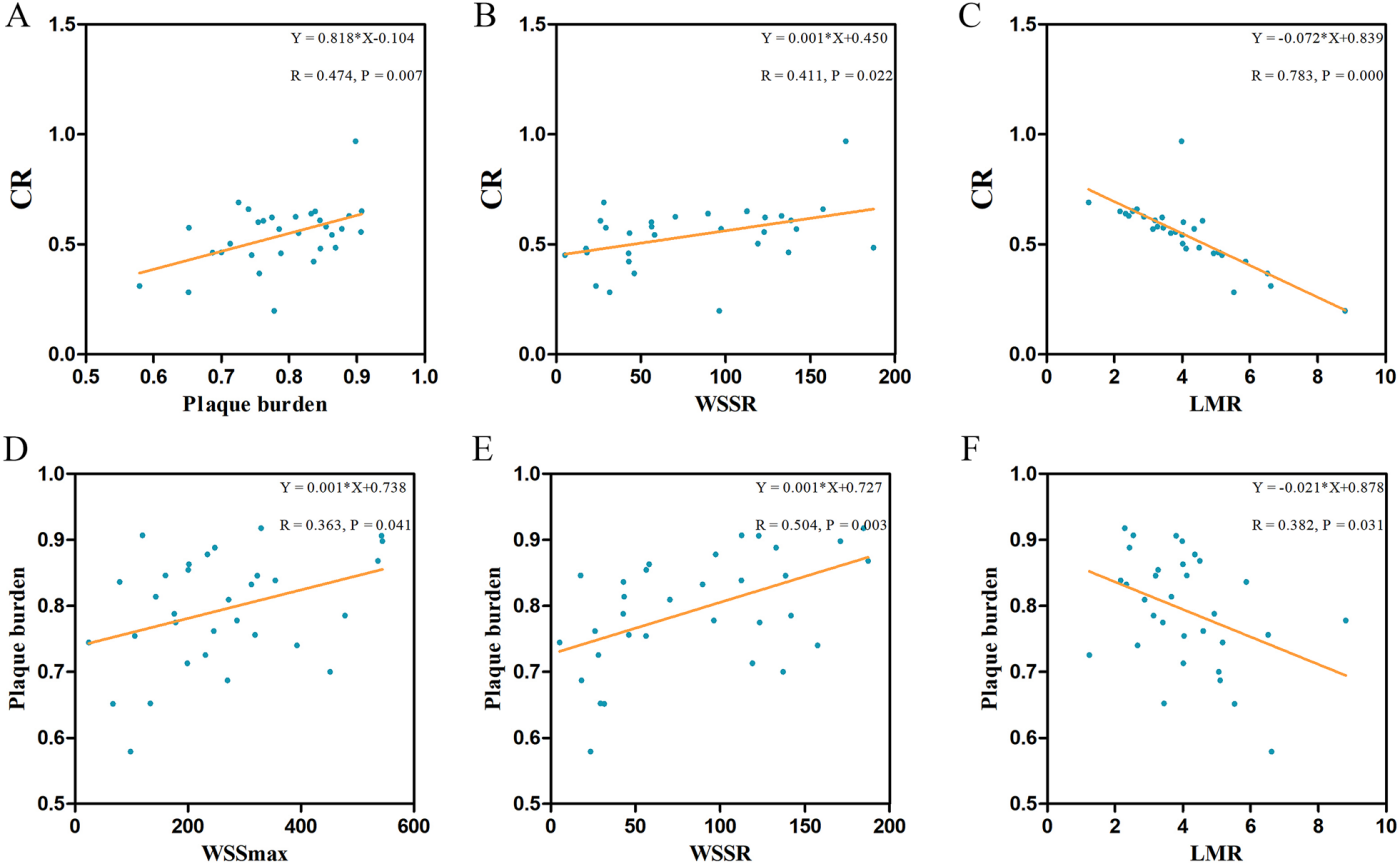
**A–C** Linear correlation between the CR with plaque burden, the WSSR, and the LMR. **D–F** Linear correlation between plaque burden with the WSSmax, the WSSR, and the LMR. The linear regression equations and correlation coefficient *R* values were provided

We constructed linear regression models and found a linear positive correlation between the CR with both the WSSR (*R* = 0.411, *P* = 0.022) and plaque burden (*R* = 0.474, *P* = 0.007), and a linear negative correlation between the CR and LMR (*R* = 0.716, *P* < 0.001) (Fig. 3A–C). In addition, the results revealed a linear positive correlation between the plaque burden with both the WSSR (*R* = 0.504, *P* = 0.003) and WSSmax (*R* = 0.363, *P* = 0.041), and a linear negative correlation between the plaque burden and LMR (*R* = 0.382, *P* = 0.031) (Fig. 3D–F).

## Discussion

Our study showed that plaque enhancement and hemodynamic parameters significantly correlated with symptomatic atherosclerotic MCA stenosis, while the WSSR was independently associated with sICAS. In addition, the WSSR was independently associated with plaque enhancement, and showed a linear positive correlation with the CR. Both the CR and the WSSR showed a linear negative correlation with the LMR. In addition, plaque burden showed a linear positive correlation with the CR, WSSmax and WSSR while showed a linear negative correlation with the LMR.

Recently, neuroinflammation has received increasing attention, HR-VWI has been used to study plaque characteristics of ICAS. In particular, previous studies have shown that ICAS plaque with enhancement on HR-VWI was unstable and that plaque enhancement was significantly associated with the occurrence of both first and recurrent ischemic stroke [22–25]. The present study also showed that qualitative and quantitative plaque enhancement on HR-VWI were significantly correlated with the symptomatic atherosclerotic MCA stenosis. Moreover, hemodynamic parameters are regarded as critical factors in ICAS plaque instability. Tuenter et al. [26] found that locally high WSS was significantly associated with intraplaque hemorrhage and calcification of carotid plaques, which can lead to reduced stability of atherosclerotic plaques. The WSSR plays a crucial role in the remodeling and plaque enlargement in the MCA atherosclerotic stenosis, and the plaques of positively remodeled vessels were unstable and tended to induce stroke [27]. Leng et al. [14] found that high WSSR and low PR were independent risk factors for recurrent ischemic stroke. They also found that high WSSR, WSSmax and low PR were significantly associated with symptomatic atherosclerotic stenosis of the MCA, and high WSSR was an independent risk factor for sICAS. Meanwhile, our previous study showed that the LMR was significantly associated with sICAS and a LMR ≤ 4.0 may be a marker of plaque instability [12]. In the present study, we found that LMR have a significant difference between enhancing and non-enhancing plaques; however, it does not have a significant difference between symptomatic and asymptomatic plaques, which may be due to the small sample size, as we also found the lowering trend of LMR in the sICAS group. Moreover, we found that the proportion of the LMR ≤ 4.0 in sICAS was higher than that in asymptomatic ICAS. The results of the present study showed that qualitative and quantitative plaque enhancement, hemodynamic parameters, and the LMR ≤ 4.0 were significantly associated with sICAS, and we hypothesized that both local and systemic inflammation as well as hemodynamics may be involved in the instability of ICAS plaques.

Our correlation analysis of the plaque characteristics, hemodynamic parameters and LMR revealed that both the CR and plaque burden showed a linear positive correlation with the WSSR, and a linear negative correlation with the LMR. In addition, our study found that WSSR was significantly associated with the LMR ≤ 4.0, indicating a significant relationship between hemodynamics and inflammation in peripheral blood. Previous studies revealed that plaque enhancement is a local inflammatory manifestation of the plaque, and plaques with a high degree of enhancement have more macrophage infiltration, indicating a stronger local inflammatory response [10, 28]. The current study suggests that a high WSSR induces stroke by weakening the function of the endothelium, thereby inducing local and systemic inflammatory responses, increasing plaque burden and decreasing plaque stability. In previous studies, Andelovic et al*.* found that WSS and oscillatory shear index (OSI) provide a powerful tool to monitor local aortic hemodynamics during ageing and atherosclerosis [29]. There are many good indicators of hemodynamics, such as OSI, were not explored in this study, and we will explored these indicators in further researches in the future.

The proportion of severe stenosis in the symptomatic group and plaque enhancement group was higher than that in the asymptomatic group and non-enhancement group, but the difference was not statistically significant, likely due to the small number of cases.

In this study HR-VWI, biomarkers in peripheral blood, and hemodynamic analysis were combined to determine which factors contribute to ICAS plaque instability and explore the relationships between these factors. The results indicated that plaque enhancement and plaque burden on HR-VWI were all associated with the WSSR and LMR, and they all were positively and linearly correlated with the WSSR, and negatively linearly correlated with LMR. The study may suggested that the interaction between hemodynamics, regional inflammation, and systematic inflammation promote progression of ICAS plaques. In addition, sICAS can help building models to predict mortality and prognosis after the treatment of general stroke patients as well as those with middle cerebral artery stenosis [30, 31].

This study has several limitations, among them are the following. First, this was a single-center retrospective study with some selection bias of cases, and a multicenter, prospective study is needed in the future. Second, the sample size of this study was small due to the relatively strict condition. Third, measuring errors may exist as the plaque characteristic and hemodynamic parameters were manually measured. In addition, our study treating blood as a viscous and incompressible Newtonian fluid, which may not fully represent the fluid mechanical properties of blood. Finally, a simplified CFD model was used to simulate the changes of focal cerebral hemodynamics, and since the regulation of blood flow by vessels was ignored, the hemodynamic parameters were not absolute, but rather relative.

## Conclusions

The present study is the first to combine plaque characteristics, hemodynamics, and peripheral blood inflammatory indicators to investigate the role of factors involved in sICAS development and their inter-relationships. Qualitative and quantitative plaque enhancement, focal hemodynamics, and the LMR, were found to be associated with sICAS, and the WSSR was identified as an independent risk factor for sICAS. Both CR and plaque burden were positively and linearly correlated with the WSSR, and negatively and linearly correlated with the LMR. The focal high WSSR induced the local and systemic inflammatory responses and increased plaque burden, ultimately leading to decreased plaque stability. This study provides new insights into the mechanisms of ICAS plaque formation and progression, which may contribute to prevention and treatment of ischemic stroke in sICAS.

### Supplementary Information

**Additional file 1**: Supplementary Materials.

## Data Availability

The data supporting the funding of this study are available from the corresponding author upon request.
