# Supplementary material for: Hemodynamics combined with inflammatory indicators exploring relationships between ischemic stroke and symptomatic middle cerebral artery atherosclerotic stenosis
Source: Eur J Med Res. 2023 Sep 26;28:378. doi: 10.1186/s40001-023-01344-8 (PMC10523698; doi:10.1186/s40001-023-01344-8)
Supplement: Supplementary file 1 — Additional file 1: Supplementary Materials. [file 40001_2023_1344_MOESM1_ESM.docx]

**Supplementary Materials**:

CTA parameters: the tube voltage, 100 kV; the tube current, 450 mA; and a rotation speed, 0.5 seconds. The intravenous contrast agent (100 mL of ioversol, 350 mg per milliliter) was injected at a flow rate of 4 mL/sec.

MRA parameters: TR/TE = 25ms/ 3.5ms, field of view = 190mm×200mm, matrix = 400 × 218, layer thickness = 0.5mm, number of layers = 84, layer spacing = -0.6 mm, flip angle = 20°, bandwidth = 216.6 Hz/pixel, the total scan time = 4 minutes and 32 seconds.

HR-VWI T1 VISTA parameters: TR/TE = 700 ms/35 ms; field of view = 200 mm × 251 mm; number of layers = 171; matrix = 252 × 314; flip angle = 80°; resolution = 0.8 mm × 0.8 mm × 0.8 mm; acquisition time = 4 minutes and 55 seconds.
